# Driver’s Visual Attention Characteristics and Their Emotional Influencing Mechanism under Different Cognitive Tasks

**DOI:** 10.3390/ijerph19095059

**Published:** 2022-04-21

**Authors:** Yaqi Liu, Xiaoyuan Wang, Longfei Chen, Shijie Liu, Junyan Han, Huili Shi, Fusheng Zhong

**Affiliations:** 1 School of Transportation and Vehicle Engineering, Shandong University of Technology, Zibo 255049, China; 4019030007@mails.qust.edu.cn; 2 College of Electromechanical Engineering, Qingdao University of Science & Technology, Qingdao 266000, China; chenlongfei@mails.qust.edu.cn (L.C.); 4019030012@mails.qust.edu.cn (S.L.); hanjunyan@mails.qust.edu.cn (J.H.); shihuili@qust.edu.cn (H.S.); bh136@qust.edu.cn (F.Z.); 3 Collaborative Innovation Center for Intelligent Green Manufacturing Technology and Equipment of Shandong, Qingdao 266000, China

**Keywords:** drivers, visual cognition, attention, emotion, traffic safety

## Abstract

The visual attention system is the gateway to the human information processing system, and emotion is an important part of the human perceptual system. In this paper, the driver’s visual attention characteristics and the influences of typical driving emotions on those were explored through analyzing driver’s fixation time and identification accuracy to different visual cognitive tasks during driving. The results showed that: the increasing complexity of the cognitive object led to the improvement of visual identification speed. The memory and recall process increased drivers’ fixation time to cognitive objects, and the recall accuracy decreased with the increase in time interval. The increase in the number of cognitive objects resulted in the driver improving the visual identification speed for the cognitive object at the end of the sequence consciously. The results also showed that: the visual cognitive efficiency was improved in the emotional states of anger and contempt, and was decreased in the emotional states of surprise, fear, anxiety, helplessness and pleasure, and the emotional state of relief had no significant effect on the visual cognitive efficiency. The findings reveal the driver’s visual information processing mechanism to a certain extent, which are of great significance to understand the inner micro-psychology of driver’s cognition.

## 1. Introduction

Among the many factors that are capable of explaining root causes of traffic accidents, human factors are important causes of preventable road-traffic incidents [1]. Rational control of driving behavior is an effective means to reduce human-caused traffic accidents [2]. The generation of drivers’ conscious behavior contains four stages: perception, cognition, decision making and action [3]. Among them, perception plays a decisive role in the decision making and is a key link in the generation of driving behavior. The sensory organs related to driver perception are mainly the visual organs, and more than 80% of the traffic information perceived by the driver originates from the visual channel [4,5]. The attention system guides the selective concentration of visual organs, and improves the awareness level of drivers to local stimuli [6]. The physiological structure of the visual system determines the limited breadth and depth of the driver’s visual perception of the traffic environment. Due to the limitation of attention resources, drivers often cannot process multiple cognitive activities at the same time [7]. The real traffic environment is complex and changeable, and missing key environmental information may lead to traffic accidents directly [8]. During the driving process, one of the main tasks of the driver is to allocate the limited physical and psychological resources reasonably from the spatial and temporal dimensions. Over the past decades, the rapid development of intelligent technology and the widespread implementation of Advanced Driving Assistance Systems (ADAS)s have effectively enhanced and extended the driver’s ability to perceive information in driving activities [9]. For example, the blind-spot monitoring system (BSM) is able to alert the driver of obstacles or oncoming traffic within the safety range behind. In more recent years, with the development of connected vehicle technology, it is foreseeable that drivers can obtain more comprehensive traffic environment information during driving in the future. However, it should be noted that human perception is limited (as mentioned above), and the environmental information delivered through the in-vehicle system is not always perceived and reasonably applied by drivers. Sometimes, too much environmental information provided can even disrupt the driver’s normal behavioral planning. Therefore, it is necessary to investigate the efficiency of drivers’ visual information perception under different cognitive tasks, as it is important to understand the boundaries of drivers’ information processing and to build a more intelligent and rational human–vehicle interaction system.

The physiological–psychological characteristics of drivers can show significant differences in different environments [10,11]. Except for external factors, the changes in the physiological–psychological characteristics of drivers due to individual factors also cannot be ignored [12]. Of the many individual factors, emotion is an individual’s attitude experience towards objective things and the corresponding physiological–psychological response [13]. In emotional activities, individuals not only accept the influence of stimuli on themselves, but also regulate their own responses to stimuli [14]. Drivers with different emotional states have significant differences in their feelings, preferences and needs for external stimuli, which in turn lead to changes in their perception and cognitive abilities [15]. In recent years, with the increasing of vicious traffic accidents caused by negative emotions such as road rage and anxiety, as well as the rapid development of affective computing and cognitive psychology, a growing interest has been seen in exploring the effects of driver’s emotions on driver’s physiological–psychological characteristics. Scholars in related fields have generally recognized that driving emotion is an important factor that cannot be ignored in traffic safety research [16].

In a nutshell, emotion is an important part of the human perceptual system, and the visual attention system is the basis of the driver’s information perception. It is of great significance to explore drivers’ visual attention characteristics and the influence of emotions on visual attention for improving the level of human–vehicle intelligent interaction in road traffic systems and improving road traffic safety.

## 2. Literature Review

In view of the important impact of drivers’ visual attention on traffic safety, scholars in related fields have conducted many studies on drivers’ eye movement, visual distraction, visual attention, etc. Xu, Y. et al. explored the relationship between the driver’s eye movement and the construction conflict through collecting and analyzing the driver’s eye movement data in simulated construction conflicts at different speeds [17]. Rahman, H. et al. presented five machine learning models and three deep learning architectures to classify a driver’s cognitive load based on driver’s eye movement signals [18]. Onkhar, V. et al. studied the effect of drivers’ eye contact on pedestrians’ perceived safety to cross the road, and demonstrated how drivers’ eye contact affects pedestrians’ perceived safety as a function of time in a dynamic scenario [19]. Li, N. and Busso, C. defined regression models with elastic net regularization and binary classifiers to separately estimate the cognitive and visual distraction levels and proposed a novel joint visual–cognitive distraction space to characterize driver behaviors [20]. Karthaus, M. et al. investigated the effects of acoustic and visual distracting stimuli on responses to critical events. The results demonstrate the high impact of distraction on driving performance in critical traffic situations and indicate a driving-related inhibition deficit in young and old drivers [21]. For the take-over of automated driving systems, the percentage of face orientation to distraction area and time to boundary at take-over timing were proposed by Li, Q. et al. to accurately evaluate the degree of visual distraction based on merely face orientation under naturalistic non-driving related tasks and to evaluate take-over performance, respectively [22]. Grahn, H. and Kujala, T. argued that visual distraction by secondary in-car tasks is a major contributing factor in traffic incidents and studied the effects of touch screen size, user interface design and subtask boundaries on in-car tasks’ visual demand and visual distraction potential [23]. Reimer, B. et al. assessed the sensitivity of visual attention and driving performance for detecting changes in driver cognitive workload across different age groups. The results showed that the degree of gaze concentration with added cognitive demand is not related to age and the driving performance measures did not show a consistent relationship with the objective demand level [24]. Muñoz, M. et al. investigated distinguishing patterns in drivers’ visual attention allocation and the results suggested that differences in glance allocation strategies serve as an effective evaluator of the visual demand of a vehicle interface [25]. Louw, T. and Merat, N. assessed drivers’ visual attention distribution during automation and on approach to a critical event, and examined whether such attention changes followed repeated exposure to an impending collision [26]. Lemonnier, S. et al. focused on three top-down factors that influence the collection of visual information: the value of visual information for the ongoing task, their bandwidth and the familiarity with the environment. Effects were found for each of the three factors in agreement with Wickens’ theoretical framework and with previous studies [27]. Young, K. et al. examined the nature of observable visual and/or manual secondary task interruptions in real-world driving. It was found that drivers interrupt only a small percentage of the secondary tasks they were engaged in, and the number of interruptions made to secondary tasks was found to differ according to some task characteristics [28]. Liu, Q. et al. tested the driver’s visual parameters when the vehicles run under the pothole repair environment and the results showed that psychology and the gaze frequency, gaze duration and saccade speed of drivers on pothole sections were significantly increased while the saccade range was reduced [29].

The driving emotion was concerned and taken as an important research object since the phenomenon that the drivers in malignant emotional states prefer to choose aggressive driving behavior, which would more likely lead to traffic accidents [30]. Further studies showed that driving emotions are inextricably linked to many risk-related factors [31]. When it comes to driving emotions, the most work in this area was oriented toward the driving emotion generation mechanism, the driving emotion recognition, and the impacts of emotions on drivers’ physiological and psychological characteristics. Barnard, M and Chapman, P. studied the relations of fear, trait anxiety, physiological and attentional reactions to accident risk. Analysis of the data suggested that fear increased with increasing accident risk, the eye movements indicated different patterns of performance according to different dangerous situations and the trait anxiety was only associated with higher rates of disliking driving and use of maladaptive coping mechanisms on questionnaires [32]. Roseborough, J. and Wiesenthal, D. examined the effect of various punishments (i.e., police enforcement, collision with a roadside object, collision with another vehicle, collision with a roadside object and police enforcement, collision with other vehicle and police enforcement) on witnesses’ feelings of anger and happiness on roadways. Analyses indicated that perceived punishment by police reduced feelings of anger and increased feelings of happiness compared to the other four forms of punishment [33]. Paschero, M. et al. proposed an Emotion Recognition System based on classical neural networks and neuro-fuzzy classifiers. In comparison with Multi-Layer Perceptron trained by EBP algorithm, the proposed Neuro-fuzzy classifiers showed very short training times, allowing applications with easy and automated setup procedures [34]. Wang, X. et al. established an online identification model for the driving emotions of joy, anger, sadness and fear based on the factor analysis method, the fuzzy comprehensive evaluation and the PAD emotional model [35]. Fairclough S and Dobbins C found that an ensemble classification model provided an accuracy rate of 73.12% for the binary classification of episodes of high vs. low anger based upon a combination of features derived from driving (e.g., vehicle speed) and cardiovascular psychophysiology (heart rate, heart rate variability, and pulse transit time) [36]. Chan, M. and Singhal, A. explored the behavioral and event-related potential (ERP) effects elicited by auditory presented words of different emotional valence during driving (dual-task) and non-driving (single-task) conditions. The results demonstrate that emotion-related auditory distraction can differentially affect driving performance depending on the valence of the emotional content [37]. Wang, X. et al. used multiple-electrocardiogram (ECG) feature fusion to recognize the driver’s emotion. Based on the back-propagation network and the Dempster–Shafer evidence method, the proposed model can recognize drivers’ anxiety with an accuracy rate of 92.89% [38]. Kadoya, Y. et al. examined the association between the taxi drivers’ on-duty emotional states and driving speed in real driving situations. The results revealed that negative emotions of taxi drivers (angry and sad) have significant impacts on increasing driving speed, a neutral emotional state is related to decreased speed, while a happy and relaxed emotional state shows no significant impact [39].

In summary, many scholars have conducted extensive and in-depth research on drivers’ visual attention and driving emotions, and have achieved fruitful research results. However, previous studies have rarely explored the driver’s visual cognitive process from the perspective of limited attentional resources, and paid less attention to the influences of emotions on drivers’ visual attention characteristics. To understand driver’s visual attention characteristics and their emotional influencing mechanism under different cognitive tasks, more systematic reviews and empirical research are required. The purpose of this study is to explore the visual attention characteristics of drivers when dealing with different cognitive tasks, and to reveal the influence mechanism of different emotions on drivers’ visual attention characteristics. This paper contains two parts of research. In study 1, the visual attention characteristics of drivers in response to different cognitive tasks were studied through designing and implementing visual identification tasks, visual working memory tasks and multiple visual identification tasks in virtual driving. In study 2, the effects of eight typical driving emotions (anger, surprise, fear, anxiety, helplessness, contempt, relief and pleasure) on the visual attention characteristics of drivers were examined based on the experimental framework of visual attention characteristic data collection proposed in Study 1 and the experimental framework of driving emotion activation and measurement proposed in our previous study [16,40].

## 3. Study 1—Driver’s Visual Attention Characteristics in Different Cognitive Tasks

### 3.1. Materials and Methods

#### 3.1.1. Participants

Sixty-seven drivers (35 males and 32 females) aged from 21 to 48 (M = 26.36, SD = 4.87) were recruited to participate in this study. All of the participants were licensed drivers and their driving experience ranged from 2 to 15 years (M = 3.93, SD = 2.84).

#### 3.1.2. Collection of Visual Attention Characteristic Data

**Figure 1:**
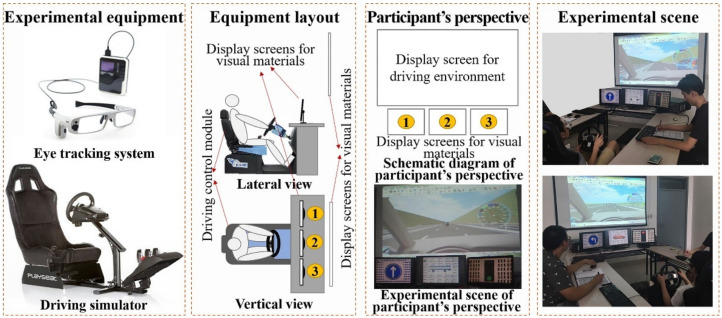
Design idea of visual attention characteristic data collection.

The experiment referred to collecting the visual attention data of the participants when they respond to different visual cognitive tasks in virtual driving through the eye-tracking system. In the experiment, the participants were required to choose any lane to drive in the virtual road environment at a speed of 80 km/h to 120 km/h on a driving simulator. The virtual driving environment was set as a two-way four-lane highway, and the single-lane traffic flow was set at 300 pcu/h. The driving simulator included a driving control module and an environment display module. The participants manipulated the vehicle through the control module, and the environment display module displayed the corresponding environmental visualization information dynamically. Three independent screens for playing visual cognitive materials were set between the driving control module and the environment display module. The participants were required to complete the visual cognitive tasks in the shortest possible time while completing driving tasks (maintaining speed and not violating traffic rules), and their visual attention data were captured and recorded by the eye-tracking system. The design idea of visual attention characteristic data collection is shown in Figure 1.

**Figure 2:**
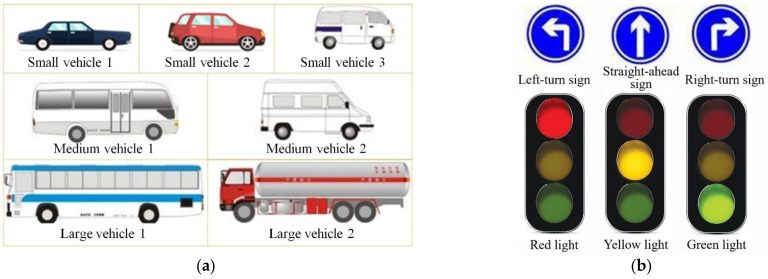
Visual cognitive materials: (**a**) Schematic diagram of vehicle types; (**b**) Schematic diagram of traffic signs and traffic lights.

Visual cognitive tasks included the Visual Identification Task (VIT), Visual Working Memory Task (VWMT) and Multiple Visual Identification Task (MVIT). The VIT was to examine visual attention time and identification accuracy when faced with a single cognitive object of varying information capacity. The VWMT was to examine visual attention time and identification accuracy when faced with a single cognitive object of varying information capacity. The MVIT was to examine visual attention allocation characteristics and identification accuracy when faced with multiple cognitive objects. In the VIT, the visual cognitive materials were static pictures randomly combined with the diagrams of different types of vehicles (Figure 2a). Each vehicle diagram contained in the visual perception picture was defined as a basic information unit, and the pictures containing 3, 5 and 7 basic information units were defined as ternary pictures, quintuple pictures and seven-element pictures, respectively. The VWMT included Visual Identification and Memory Task (VIMT) and Visual Identification and Recall Task (VIRT). A VIMT and a VIRT constituted a Memory Task Unit (MTU). In the VWMT, the visual cognitive materials were all seven-element pictures composed of vehicle diagrams. In the MVIT, the visual cognitive materials were played simultaneously on the three independent screens, and the content played on each screen was a schematic diagram of a traffic sign, a signal light or a vehicle mode in Figure 2, respectively. Each traffic sign or signal light schematic included in the picture was also identified as a basic information unit, and the visual materials played in the MVIT were all unary information pictures. The materials played on the three screens at one time were a random combination of a traffic sign, a signal light and a vehicle model diagram, and the content attributes of the pictures played on each screen are different (that is, when the traffic signs were played on screen 1, the other two screens will no longer select the traffic signs, and so on).

**Figure 3:**
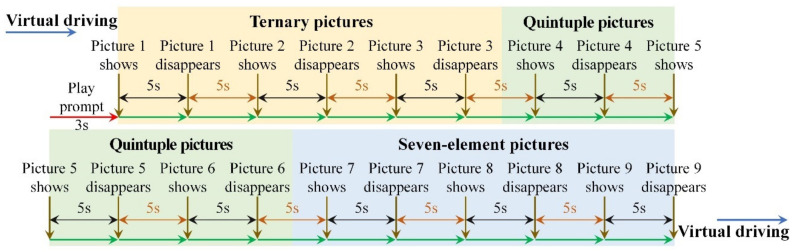
Display timeline of visual cognitive materials in VIT.

**Figure 4:**
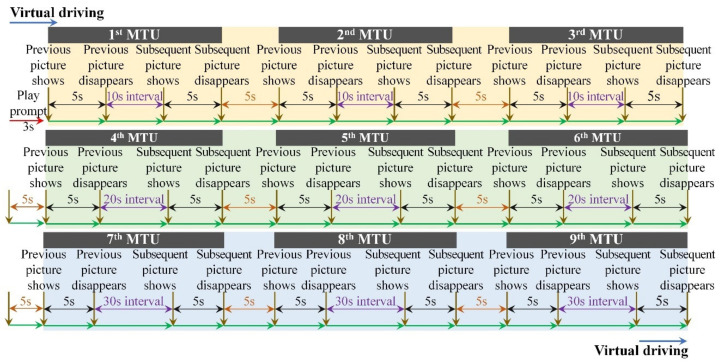
Display timeline of visual cognitive materials in VWMT.

**Figure 5:**
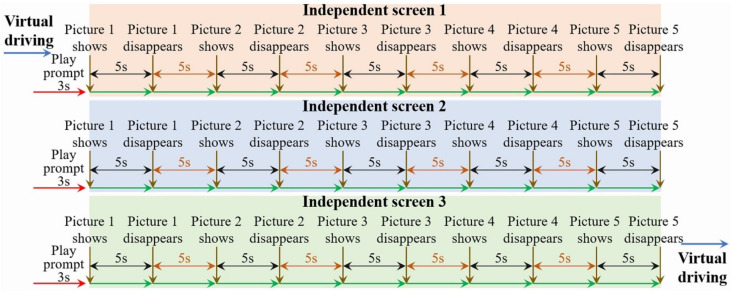
Display timeline of visual cognitive materials in MVIT.

A further elaboration on the different visual cognitive tasks is shown in Table 1, and the display timeline of visual cognitive materials in VIT, VWMT and MVIT are shown in Figure 3, Figure 4 and Figure 5, respectively.

**Figure 6:**
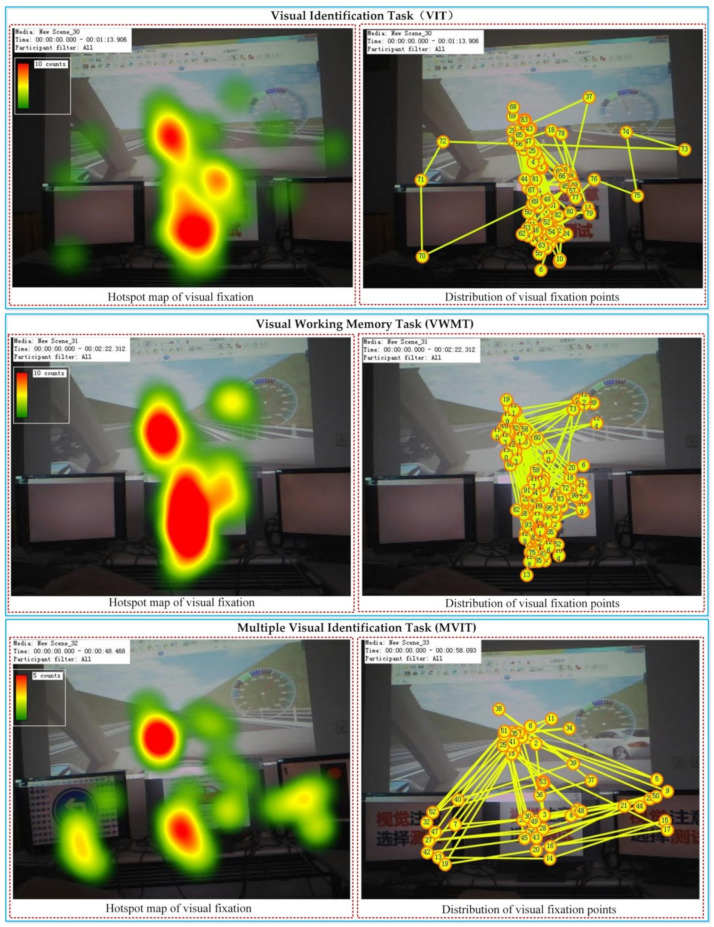
Fixation point distribution of a participant. Note: In the “Distribution of visual fixation points”, each dot represents a visual fixation point. The numeric code represents the temporal order of the fixation points, and the two fixation points adjacent to each other in the temporal order are connected by a straight line.

Before the visual attention characteristic data collection, the participants were trained to be proficient in the operation of the driving simulator and to understand the meaning of the vehicle models, traffic signs and signal lights in Figure 2. For each participant, the three kinds of visual tasks were performed in sequence, and the interval between each kind of task was set to 15 s. During this interval, the participants did not interrupt the virtual driving, and the duration of each visual attention characteristic data collection was about 10 min. In the experiment, the eye-tracking system was used to record the distribution of the participants’ fixation points in real time. The fixation point distribution of a participant in the experiment is shown in Figure 6.

#### 3.1.3. Data Preprocessing

The visual characteristic data obtained from the experiment were imported into the data analysis software of the eye-tracking system. The data analysis software can display the fixation index of the participants at any time, and each fixation point represents 0.033 s of fixation duration. The driving simulator environment display screen and the independent screens 1 to 3 are demarcated as Area of Interest (AOI) 1 to 4, respectively. The duration of the participants’ fixation on the visual cognitive materials was obtained through counting the number of fixation points in each AOI in a specific time period. The identification accuracy of different visual materials was obtained by counting the picture identification results reported by the participants. The explanatory notes of the relevant parameters (symbols) obtained from the experiments are shown in Table 2.

### 3.2. Results and Discussions

#### 3.2.1. Visual Attention Characteristics in VIT

**Figure 7:**
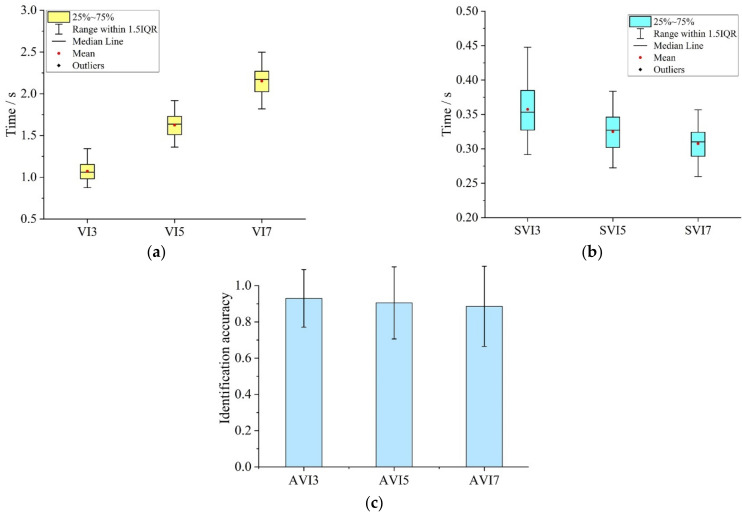
Drivers’ visual attention characteristics in VIT: (**a**) Fixation time for visual tasks; (**b**) Fixation time for basic information unit; (**c**) Identification accuracy for visual tasks.

Figure 7 shows the fixation time and identification accuracy of the participants in the VIT for multi-information pictures. As shown in Figure 7a, as the number of the basic information units in the picture increases, the fixation time increases synchronously. The statistical results showed that the value range of VI3 was 0.875~1.343 s (M = 1.072, SD = 0.117), the value range of VI5 was 1.362~1.919 s (M = 1.624, SD = 0.143) and the value range of VI7 was 1.819~2.500 s (M = 2.153, SD = 0.161). As shown in Figure 7b, with the increase in the number of basic information units in the picture, participants’ fixation time to a single basic information unit decreased. The statistical results showed that the value range of SVI3 was 0.292~0.448 s (M = 0.357, SD = 0.039), the value range of SVI5 was 0.272~0.384 s (M = 0.325, SD = 0.029) and the value range was 0.260~0.357 s (M = 0.308, SD = 0.023). Figure 7c showed the average identification accuracy for different pictures (AVI3, AVI5 and AVI7) in the VIT. The statistical results showed that the value range of the identification accuracy rates was 0.333~1. Among them, AVI3, AVI5 and AVI7 were 0.930 (SD = 0.159), 0.905 (SD = 0.199) and 0.886 (SD = 0.221), respectively.

One-way ANOVA was performed on SVI (Table 3). The results showed significant differences among SVI3, SVI5 and SVI7 (F = 45.172). Further, the results of multiple comparison analyses of SVI showed that there were significant differences between SVI3, SVI5 and SVI7 pairwise (Table 4). The above results demonstrated that the participants’ fixation time for a single basic information unit in a multi-information picture decreases with the increase in the number of basic information units. These results should be related to the fact that the participants performed virtual driving while completing the visual identification task. The virtual driving task simulated real driving activities and required participants not to focus their visual attention on a single cognitive object for a long time. As the complexity of the visual identification task increases, the participants would consciously improve the visual identification processing speed of a single cognitive object, thereby reducing the average attention time of the corresponding basic information unit.

One-way ANOVA was performed on the identification accuracy of ternary, quintuple and seven-element pictures (AVI). The results (Table 5) showed no significant difference in the identification accuracy of different pictures (F = 0.886), but the identification accuracy showed a downward trend with the increase in picture information units.

#### 3.2.2. Visual Attention Characteristics in VWMT

**Figure 8:**
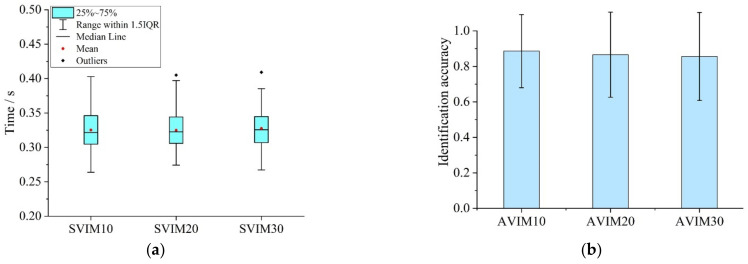
Drivers’ visual attention characteristics in VIMT: (**a**) Fixation time for basic information unit; (**b**) Identification accuracy for visual tasks.

The driver’s memory in the driving process is a typical working memory [41,42]. The VWMT aimed at stimulating the driver’s working memory process, and the VIMT corresponded to the information input stage, and VIRT corresponded to the information extraction and identification stage in the working memory process. Figure 8 shows the fixation time and identification accuracy for seven-element pictures in the VIMT.

The visual materials in the VIMT were all seven-element pictures. Figure 8a shows the participants’ fixation time (SVIM) for a single basic information unit in the VIMT. The statistical results showed that the range of SVIM10 was 0.264~0.403 s (M = 0.325, SD = 0.028), the range of SVIM20 was 0.264~0.403 s (M = 0.325, SD = 0.027) and the range of SVIM30 was 0.267~0.409 s (M = 0.328, SD = 0.028). The results of one-way ANOVA on SVIM (Table 6) showed that there was no significant difference among SVIM10, SVIM20 and SVIM30.

Figure 8b shows the average identification accuracy of the visual materials (AVIM) in the VIMT. The statistical results showed that the value of AVIM ranged from 0.333 to 1. Among them, when the display interval of the previous and subsequent picture was 10 s, the average identification accuracy (AVIM10) was 0.886 (SD = 0.206). When the display interval of the two pictures was 20 s, the average identification accuracy (AVIM20) was 0.866 (SD = 0.240). When the display interval of the two pictures was 30 s, the average identification accuracy (AVIM30) was 0.856 (SD = 0.248). The results of one-way ANOVA (Table 7) showed that there was no significant difference between AVIM10, AVIM20 and AVIM30 (F = 0.288).

In the VIMT, participants were required to identify and memorize visual materials. This kind of memory was conscious memory, which required the participants to make a certain volitional effort [43]. In the VIT, the fixation time (SVI7) of the participants to the basic information unit in the seven-element picture was 0.308 s (SD = 0.023). In the VIMT, SVIM10, SVIM20 and SVIM30 were 0.325 s (SD = 0.028), 0.325 s (SD = 0.027) and 0.328 s (SD = 0.028), respectively. The one-way ANOVA results (Table 8) showed a significant difference between SVI7 and SVIM (F = 8.159). The results of multiple comparison analysis (Table 9) showed that SVI7 was different from SVIM10, SVIM20 and SVIM30, significantly. This indicated that the memory process increased the participants’ fixation time on visual materials significantly.

In the VIT, AVI7 was 0.886 (SD = 0.221). In the VIMT, AVIM10, AVIM20 and AVIM30 were 0.885 (SD = 0.201), 0.866 (SD = 0.240) and 0.856 (SD = 0.248), respectively. The one-way ANOVA results showed (Table 10) no significant difference between AVI7 and AVIM (F = 0.284); that is, the memory process did not affect the identification accuracy.

**Figure 9:**
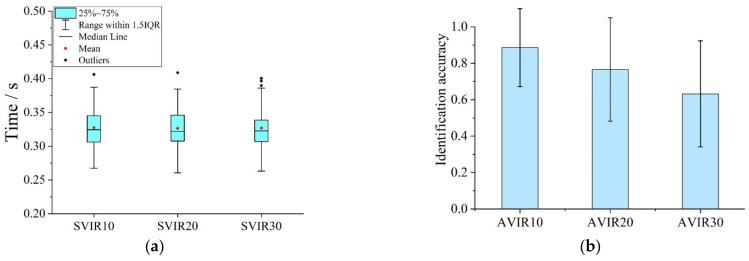
Drivers’ visual attention characteristics in VIRT: (**a**) Fixation time for basic information unit; (**b**) Identification accuracy for visual tasks.

Figure 9a shows the average fixation time of the participants to the basic information unit in the VIRT (SVIR). The statistical results showed that the value range of SVIR10 was 0.267~0.406 s (M = 0.328, SD = 0.027), the value range of SVIR20 was 0.261~0.409 s (M = 0.326, SD = 0.029) and the value range of SVIR30 was 0.263~0.401 s (M = 0.327, SD = 0.029). One-way ANOVA results (Table 11) showed that there was no significant difference among SVIR10, SVIR20 and SVIR30 (F = 0.016).

As shown in Figure 9b, with the increase in the display time interval between the previous and subsequent picture, AVIR kept decreasing. The value range of AVIR10, AVIR20 and AVIR30 was 0.333~1 (M = 0.886, SD = 0.214), 0~1 (SD = 0.284) and 0~1 (M = 0.766, SD = 0.291), severally. One-way ANOVA (Table 12) and multiple comparison analysis (Table 13) showed significant differences between pairs of AVIR10, AVIR20 and AVIR30. According to [44], the correct rate of recall drops to about 10% after 18 s. The recall accuracy for visual information in this experiment declined more slowly over time. After the visual information disappeared for 10 s, 20 s and 30 s, the recall accuracy was 88.6%, 76.6% and 63.2%, respectively.

In the VIT, SVI7 was 0.308 s (SD = 0.023). In the VIRT, SVIR10, SVIR20 and SVIR30 were 0.328 s (SD = 0.027), 0.326 s (SD = 0.029) and 0.327 s (SD = 0.029), respectively. One-way ANOVA (Table 14) and multiple comparison analysis (Table 15) showed significant differences between SVI7 and SVIR. It was proved that the recall matching process increases participants’ fixation time to visual materials.

In the VIT, the identification accuracy for seven-element pictures (AVI7) was 0.886 (SD = 0.221). In the VIRT, AVIR10, AVIR20 and AVIR30 were 0.886 (SD = 0.214), 0.766 (SD = 0.318) and 0.632 (SD = 0.291), respectively. The multiple comparison analysis results of AVI7, AVIR10, AVIR20 and AVIR30 (Table 16) showed that AVI7 was significantly different from AVIR20 and AVIR30, but not from AVIR10. It indicated that the recall accuracy in VIRT began to decline after the visual information disappeared 10 s.

In VIMT and VIRT, the properties of the visual materials were the same, but the cognitive tasks that the participants need to complete were different. In order to examine the effects of different cognitive tasks on the participants’ fixation time, paired-sample *T*-tests were performed on SVIM10 and SVIR10, SVIM20 and SVIR20, and SVIM30 and SVIR30, respectively. The results showed (Table 17) no significant difference between the above-paired variables. It suggested that there was no significant difference in the effects of the two visual cognitive task attributes on the participants’ fixation time.

#### 3.2.3. Visual Attention Characteristics in MVIT

**Figure 10:**
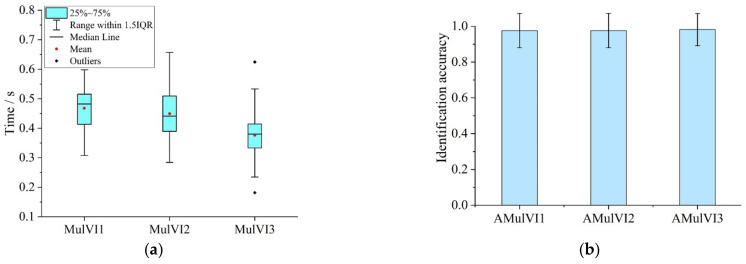
Drivers’ visual attention characteristics in MVIT: (**a**) Fixation time for basic information unit; (**b**) Identification accuracy for visual tasks.

In the MVIT, participants were required to identify the visual materials played simultaneously on screens 1 to 3. Figure 10a shows the average fixation time to unary pictures on each screen (MulVI). The statistical results showed the range of fixation time on the unary picture in screen 1 (MulVI1) was 0.307~0.598 s (M = 0.468, SD = 0.070), the fixation time on the unary picture in screen 2 (MulVI2) ranged from 0.284 to 0.657 s (M = 0.449, SD = 0.081) and the value range for the fixation time on the unary picture in screen 3 (MulVI3) was 0.182 to 0.625 s (M = 0.376, SD = 0.083).

The above results showed that MulVI3 was smaller than MulVI1 and MulVI2, distinctly. One-way ANOVA and multiple comparison analysis results (Table 18 and Table 19) showed that MulVI3 was significantly different from both MulVI1 and MulVI2. This result indicated that when there are a large number of visual tasks to be processed, drivers would consciously improve the identification speed of the cognitive object at the end of the sequence, and then reduce the fixation time of the corresponding visual task. Therefore, when dealing with multiple visual cognitive objects at the same time, the drivers’ attention time on the cognitive object at the end of the sequence would decrease with the compression of disposable time until an Attentional Blink (AB) occurs [45].

Figure 10b shows the identification accuracy of visual materials in the MVIT(AMulVI). The AMulVI1, AMulVI2 and AMulVI3 were 0.976 (SD = 0.096), 0.976 (SD = 0.096) and 0.982 (SD = 0.090), respectively. The one-way ANOVA (Table 20) showed there was no significant difference among AMulVI1, AMulVI2 and AMulVI3 (F = 0.090).

## 4. Study 2—Influences of Emotions on Driver’s Visual Attention Characteristics

### 4.1. Materials and Methods

#### 4.1.1. Participants

Forty-three drivers (35 males and 32 females) with normal vision were selected through social recruitment to participate in the study. The age distribution of the participants was 20 to 40 years old (M = 27.53, SD = 4.90). All the participants were licensed drivers and the driving experience was distributed from 1 to 12 years (M = 4.67, SD = 2.57).

#### 4.1.2. Collection of Visual Attention Characteristics Data in Different Emotional States

The purpose of this experiment was to collect data on the visual attention characteristics of participants in a neutral emotional state and eight typical emotional states. The main steps of the experiment included emotion activation, collection of visual attention characteristic data and evaluation of emotion activation efficacy.

1. Emotion activation

The neutral emotional state in this study referred to a state of mind in which the mood is calm and without any emotional swing. For the neutral state activation, the participants were asked to listen to a piece of soothing music before the start of the relevant test, and then participated in each test according to their personal habits and behavioral styles. For the other emotions, the emotion activation methods referred to the literature [20,29]. The activation of each emotion included primary activation and deep activation. The methods used in the primary activation included picture activation, reward activation, personal recall activation and competitive game. On the basis of the primary activation, the deep activation was carried out, and the method used was the video activation.

2. Collection of visual attention characteristic data

**Figure 11:**
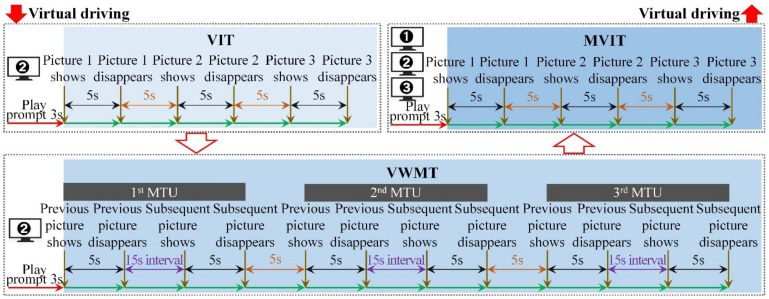
Visual materials display timeline of each visual attention characteristic data collection.

The general idea of visual attention characteristics data collection was similar to Section 3.1.2 in study 1, but the experiments were simplified considering the timeliness of emotion activation efficacy. The experiment included visual cognitive tasks included the Visual Identification Task (VIT), Visual Working Memory Task (VWMT) and Multiple Visual Identification Task (MVIT). Each participant was required to complete a visual attention characteristic data collection under a neutral state and eight typical emotional states, respectively. In each visual attention characteristic data collection, the VIT, VWMT and MVIT were performed, sequentially, and the interval between the three types of tasks was 15 s. During this interval, the participants did not interrupt the virtual driving. In a single VIT, a total of 3 seven-element pictures were displayed on screen 2. In a single VWMT, a total of 3 sets of seven-element pictures (6 pictures in total) were played on screen 2. The participants were asked to identify and report whether the proportions of vehicle models contained in the previous and subsequent pictures were the same in the shortest possible time. In a single MVIT, screens 1, 2 and 3 displayed 3 groups of 9 pictures containing the vehicle types, traffic signs, and traffic light schematic diagrams (Figure 2) simultaneously. Figure 11 shows the visual materials display timeline of the single visual attention characteristic data collection in Study 2.

3. Evaluation of emotion activation efficacy

Emotion activation efficacy evaluation was performed after the visual attention characteristics data collection. The overall idea was referred to [20,29], and the measurement tool used was the PAD scale (Figure S1 in Supplementary Materials). Before conducting relevant experiments, the connotation of the PAD scale was explained to the participants to ensure that they could use the scale to accurately describe their minds. After each visual attention characteristic data collection, the participants filled in the PAD scale once. The emotional state filled in the PAD scale corresponded to a point in the PAD space. The Euclidean distance between this point and the emotion coordinate was used to represent the activation strength of the corresponding emotion [24]. For example, the anxiety state filled in by a participant was (3, 2, 6), which corresponds to the point (−0.5, −0.75, 0.25) in the PAD space. The distance between the point (−0.5, −0.75, 0.25) and the coordinates of anxiety in the PAD space (−0.24, 0.08, −0.16) represented the activation efficacy of anxiety. The smaller the distance was, the higher the activation efficiency of anxiety was. The PAD scale can represent a total of 729 emotional states [20,29], corresponding to 729 points in the PAD space. The distances between the 729 points and the coordinates of 8 typical emotions were sorted, and the corresponding emotional activation efficacy of each point was assigned according to the distance distribution (Table S1 in Supplementary Materials). The assignment range was 0~5. The larger the value was, the higher the activation efficiency of the corresponding emotion was.

#### 4.1.3. Data Preprocessing

The visual data obtained in the above experiments were imported into the data analysis software of the eye-tracking system, and the fixation time of the participants on each visual material was obtained. The identification accuracy of different visual materials was obtained by counting the picture identification results reported by the participants. Based on the above method, the visual attention characteristics data of 43 participants under a neutral state and 8 typical emotions were obtained. The average fixation time of participants to 3 seven-element pictures in a single VIT was denoted as VI’. The average identification accuracy of 3 seven-element pictures in a single VIT was recorded as AVI’. The average fixation time of participants to 3 subsequent pictures in a single VWMT was denoted as VIR’. The average identification accuracy of 3 subsequent pictures in a single VWMT was recorded as AVIR’. It should be pointed out that the fixation time and identification accuracy of the previous pictures in VWMT were no longer recorded and analyzed. The average fixation time of participants to 9 unary pictures in a single MVIT was recorded as MulVI’. The average identification accuracy of 9 unary pictures in a single MVIT was recorded as AMulVI’.

### 4.2. Results and Discussion

**Figure 12:**
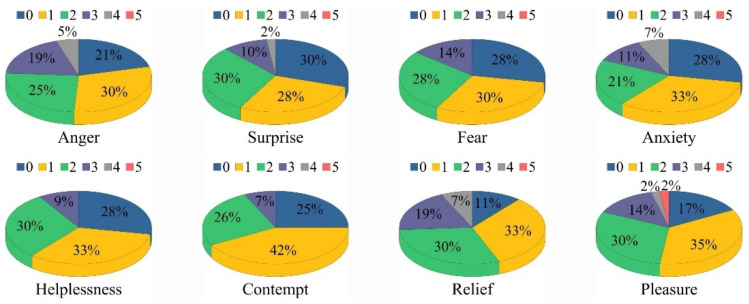
Proportion of activation efficacy levels of eight typical emotions.

**Figure 13:**
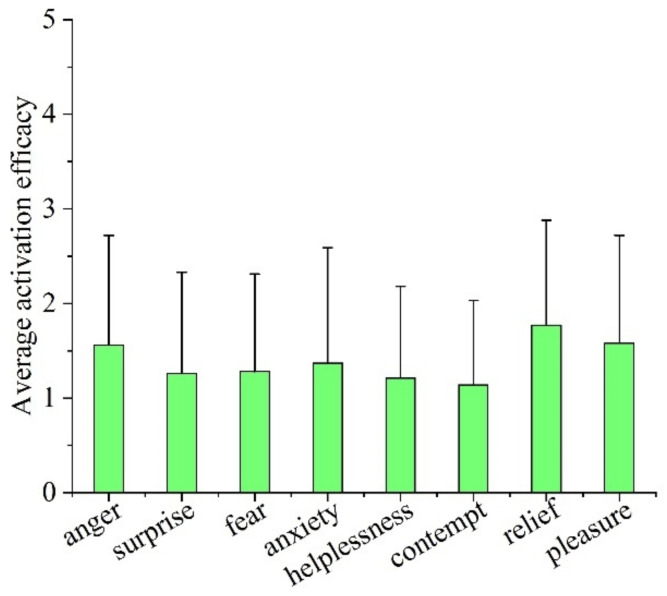
Average activation efficacy of eight typical emotions.

Figure 12 shows the proportion of activation efficacy levels for eight typical emotions, and Figure 13 shows the average activation efficacy of the emotions. According to statistics, the average activation efficacy of anger, surprise, fear, anxiety, helplessness, contempt, relief and pleasure were 1.56 (SD = 1.16), 1.26 (SD = 1.07), 1.28 (SD = 1.03), 1.37 (SD = 1.22), 1.21 (SD = 0.97), 1.14 (SD = 0.89), 1.77 (SD = 1.11) and 1.58 (SD = 1.14), respectively.

#### 4.2.1. Influences of Different Emotions on Visual Attention Characteristics in VIT

**Figure 14:**
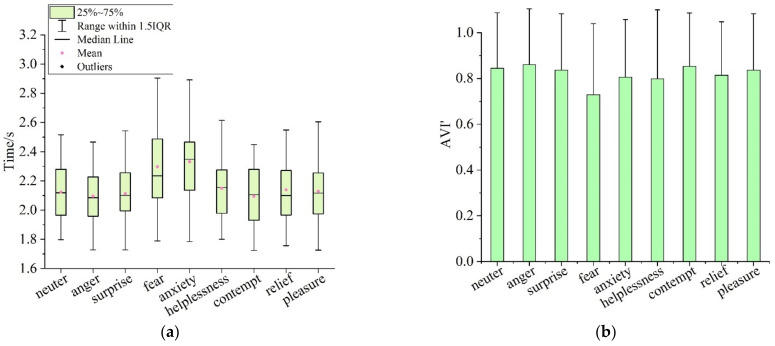
Visual attention characteristics of participants with different emotional states in VIT: (**a**) fixation time on visual materials; (**b**) identification accuracy of visual materials.

Figure 14 shows the visual attention characteristics of participants in different emotions in the VIT. Figure 14a,b were the fixation time (VI’) and identification accuracy (AVI’) to visual materials, severally. According to statistics, the average fixation time of the participants in the states of neuter, anger, surprise, fear, anxiety, helplessness, contempt, relief and pleasure to the visual materials was 2.124 s (SD = 0.199), 2.095 s (0.193), 2.114 s (SD = 0.202), 2.297 s (SD = 0.277), 2.332 s (SD = 0.255), 2.150 s (SD = 0.219), 2.095 s (SD = 0.204) and 2.129 s (SD = 0.217), respectively, the average identification accuracy of visual materials was 0.845 (SD = 0.245), 0.860 (SD = 0.244), 0.837 (SD = 0.245), 0.729 (SD = 0.311), 0.806 (SD = 0.254), 0.798 (SD = 0.301), 0.853 (SD = 0.233), 0.814 (SD = 0.233) and 0.837 (SD = 0.245), severally.

In order to further test the influences of different emotions on visual attention characteristics in the VIT, the VI’ and AVI’ of the participants in the neutral state and in eight emotional states were tested by paired samples *T*-test (Table 21 and Table 22). According to the *T*-test results, there was a significant difference between the VI’ of participants in the angry state and neutral state (t = 4.416), and there was no significant difference between the AVI’ of participants in the angry state and neutral state (t = −1.431). It suggested that without affecting the identification accuracy of visual materials, the fixation time of angry participants on visual materials was significantly reduced; that is, the emotional state of anger improved the cognitive efficiency on visual materials in the VIT. The VI’ (t = −6.363) and AVI’ (t = 4.743) of participants in the fearful state were significantly different from those of participants in the neutral state. It showed that the fearful participants’ fixation time on visual materials increased significantly, but the identification accuracy of visual materials decreased significantly; that is, the cognitive efficiency of the fearful participants was significantly declined in the VIT. There was a significant difference between the VI’ of participants in the anxious state and neutral state (t = −7.449), and there was no significant difference between the AVI’ of participants in the anxious state and neutral state (t= 1.703). This indicated that the participants in the anxiety state spend more fixation time on visual material, while did not improve the identification accuracy; that is, the emotional state of anxiety led to a decrease in the cognitive efficiency of the participants in the VIT for visual materials. The VI’ (t = −3.235) and AVI’ (t = 2.610) of the helpless participants were significantly different from those in the neutral state. It showed that the emotional state of helplessness led to an increase in the participants’ fixation time on visual materials, but reduced the identification accuracy of visual materials; that is, the visual cognitive efficiency of helpless participants in VIT was significantly lower than that of neutral subjects. There was a significant difference between the VI’ of participants in the contempt and neutral state (t = 3.695), and there was no significant difference between the AVI’ of participants in the contempt and neutral state (t = −0.330). This showed that, without affecting the identification accuracy of visual materials, subjects in the emotional state of contempt spend less fixation time on visual materials; that is, the emotion of contempt improved the cognitive efficiency of participants in the VIT. The VI’ and AVI’ of the participants in the emotional states of surprise, relief and pleasure were not significantly different from that of the participants in the neutral state, indicating that the three emotions had no significant influence on participants’ fixation time and identification accuracy of visual materials in the VIT.

#### 4.2.2. Influences of Different Emotions on Visual Attention Characteristics in VWMT

**Figure 15:**
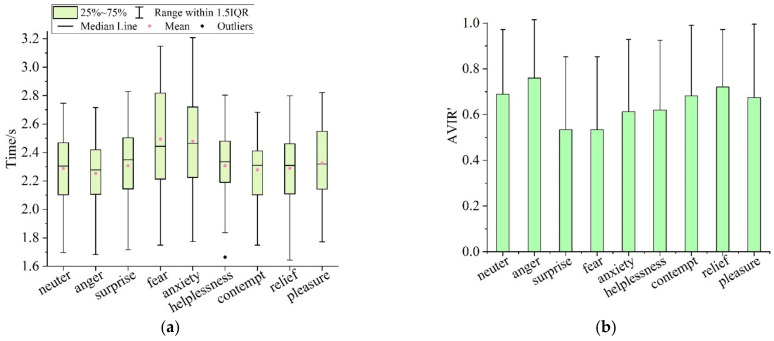
Visual attention characteristics of participants with different emotional states in VWMT: (**a**) fixation time on visual materials; (**b**) identification accuracy of visual materials.

Figure 15 shows the visual attention characteristics of participants with different emotions in the VWMT. Figure 15a,b were the fixation time (VIR’) and identification accuracy (AVIR’) to visual materials, severally. According to statistics, the average fixation time of the participants in the states of neuter, anger, surprise, fear, anxiety, helplessness, contempt, relief and pleasure to subsequent pictures was 2.288 s (SD = 0.252), 2.252 s (SD= 0.244), 2.307 s (SD = 0.259), 2.494 s (SD = 0.351), 2.479 s (SD = 0.329), 2.306 s (SD = 0.253), 2.279 s (SD = 0.237), 2.289 s (SD = 0.253) and 2.324 s (SD = 0.260), and the average identification accuracy of subsequent pictures was 0.690 (SD = 0.285), 0.760 (SD = 0.255), 0.535 (SD = 0.318), 0.535 (SD = 0.318), 0.612 (SD = 0.316), 0.620 (SD = 0.305), 0.682 (SD = 0.308), 0.721 (SD = 0.251) and 0.674 (SD = 0.321).

To further test the influences of emotions on visual attention characteristics in the VWMT, the VIR’ and AVIR’ of the participants in the neutral state and in eight emotional states were tested by paired samples *T*-test (Table 23 and Table 24). According to the *T*-test results, the VIR’ (t = 5.072) and AVIR’ (t = −3.334) of the angry participants were significantly different from those in the neutral state. This showed that the emotion of anger not only reduced the participants’ fixation time on visual materials, but also improved the participants’ identification accuracy of visual materials; that is, the motion of anger significantly improved the participants’ cognitive efficiency of each subsequent picture in the VWMT. The VIR’ (t = −2.864) and AVIR’ (t = 6.043) of the participants in the surprised state were significantly different from those in the neutral state. It showed that the emotion of surprise increased the participants’ fixation time on the visual materials, while it reduced the identification accuracy to the visual materials; that is, the emotion of surprise significantly declined the cognitive efficiency of the participants in the VWMT. In the emotional state of fear, the participants’ VIR’ (t = −7.321) and AVIR’ (t = 5.547) were significantly different from those in the neutral state. It showed that the emotion of fear not only increases the participants’ fixation time on visual materials, but also reduced the identification accuracy of visual materials; that is, the emotion of fear significantly reduced the participants’ cognitive efficiency in the VWMT. The VIR’ (t = −6.361) and AVIR’ (t = 2.673) of anxious participants were significantly different from those of participants in the neutral state. This showed that the emotion of anxiety not only increases the participants’ fixation time on visual materials, but also reduces the identification accuracy of visual materials; that is, the emotion of anxiety significantly reduced the participants’ cognitive efficiency in the VWMT. In the emotional state of helplessness, the participants’ VIR’ (t = −5.126) and AVIR’ (t = 3.334) were significantly different from those in the neutral state. This indicated that the emotion of helplessness not only increases the participants’ fixation time on visual materials, but also reduces the identification accuracy of visual materials; that is, the emotion of helplessness led to participants’ cognitive efficiency to decline significantly in the VWMT. The VIR’ (t = −5.126) of the participants in the pleasant state was significantly different from that of the participants in the neutral state, and there was no significant difference between the AVIR’ (t = 0.496) of participants in the pleasant state and in the neutral state. It showed that the emotion of pleasure increased the participants’ fixation time on visual materials, but did not affect the participants’ identification accuracy of visual materials; that is, the emotion of pleasure declined the participants’ cognitive efficiency of each subsequent picture in the VWMT. Neither the VIR’ nor the AVIR’ of the participants in the emotional states of contempt and relief was significantly different from those of participants in the neutral state. It showed that the emotions of contempt and relief did not affect the participants’ cognitive efficiency of visual materials in the VWMT.

#### 4.2.3. Influences of Different Emotions on Visual Attention Characteristics in MVIT

**Figure 16:**
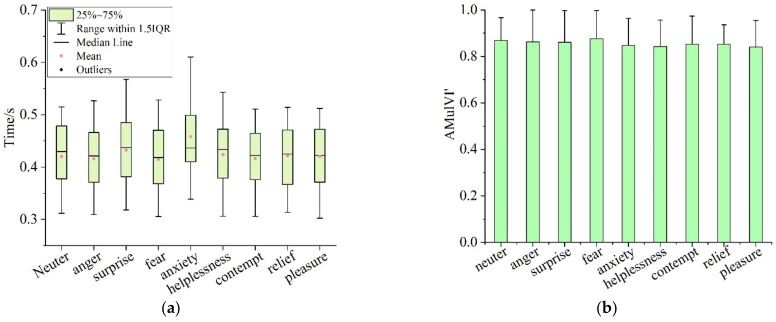
Visual attention characteristics of participants with different emotional states in MVIT: (**a**) fixation time on visual materials; (**b**) identification accuracy of visual materials.

Figure 16 shows the visual attention characteristics of participants with different emotions in the MVIT. Figure 16a,b were the fixation time (MulVI’) and identification accuracy (AMulVI’) to the visual materials, severally. According to statistics, the average fixation time of participants in the states of neuter, anger, surprise, fear, anxiety, helplessness, contempt, relief and pleasure to unary pictures was 0.420 s (SD = 0.058), 0.416 s (SD = 0.058), 0.433 s (SD = 0.064), 0.414 s (SD = 0.058), 0.458 s (SD = 0.069), 0.424 s (SD = 0.060), 0.416 s (SD = 0.056), 0.422 s (SD = 0.059) and 0.420 s (SD = 0.057), severally, and the average identification accuracy of unary pictures was 0.868 (SD = 0.098), 0.863 (SD = 0.137), 0.860 (SD = 0.137), 0.876 (SD = 0.122), 0.848 (SD = 0.116), 0.842 (SD = 0.114), 0.853 (SD = 0.120), 0.853 (SD = 0.083) and 0.840 (SD = 0.115), respectively.

To further test the influences of emotions on visual attention characteristics in the MVIT, the MulVI’ and AMulVI’ of the participants in the neutral state and in eight emotional states were tested by paired samples *T*-test (Table 25 and Table 26). According to the *T*-test results, there was a significant difference between the MulVI’ of participants in the angry state and neutral state (t = 3.398), and there was no significant difference between the AMulVI’ of participants in the angry state and neutral state (t = 0.496). It suggested that without affecting the identification accuracy of visual materials, the fixation time of angry participants on visual materials was significantly reduced; that is, the emotion of anger improved the cognitive efficiency on visual materials in the MVIT. There was a significant difference between the MulVI’ of participants in the surprised state and neutral state (t = −5.267), and there was no significant difference between the AMulVI’ of participants in the surprised state and neutral state (t = 0.573). It suggested that the emotion of surprise increased the participants’ fixation time on the visual materials, while had no significant effect on the identification accuracy; that is, the emotion of surprise significantly declined the cognitive efficiency of the participants in the MVIT. Neither the MulVI’ (t = 0.916) nor the AMulVI’ (t = −0.724) of the participants in the emotional state of fear were significantly different from those of participants in the neutral state. It showed that the emotions of fear did not affect the participants’ cognitive efficiency of visual materials in the MVIT. There was a significant difference between the MulVI’ of participants in the anxious state and neutral state (t = −6.902), and there was no significant difference between the AMulVI’ of participants in the anxious state and neutral state (t = 1.838). This suggested that the emotion of anxiety increased the participants’ fixation time to visual materials, but did not significantly change the identification accuracy of visual materials; that is, the emotion of anxiety declined participants’ cognitive efficiency of visual materials in the MVIT. In the emotional state of helplessness, the participants’ MulVI’ (t = −2.327) and AMulVI’ (t = 3.177) were significantly different from those in the neutral state. This indicated that the emotion of helplessness not only increases the participants’ fixation time on visual materials, but also reduced the identification accuracy of visual materials; that is, the emotion of helplessness led to participants’ cognitive efficiency declined significantly in the MVIT. There was a significant difference between the MulVI’ of participants in the contempt and neutral state (t = 2.333), and there was no significant difference between the AMulVI’ of participants in the contempt and neutral state (t = 1.634). It suggested that the fixation time of participants in contempt on visual materials was significantly reduced, and the emotion of contempt had no significant effect on the identification accuracy of visual materials; that is, the emotion of contempt improved the cognitive efficiency on visual materials in the MVIT. Neither the MulVI’ (t = −0.722) nor the AMulVI’ (t = 1.431) of the participants in the emotional state of relief were significantly different from those of participants in the neutral state, which suggested that the emotions of relief did not affect the participants’ cognitive efficiency of visual materials in the MVIT. There was no significant difference between the MulVI’ of participants in the pleasant state and neutral state (t = 0.114), and there was a significant difference between the AMulVI’ of participants in the pleasant state and neutral state (t = 2.886). This suggested that the emotion of pleasure had no significant effect on participants’ fixation time on the visual materials, while reduced the identification accuracy significantly; that is, the emotion of pleasure significantly declined the cognitive efficiency of the participants in the MVIT.

#### 4.2.4. Comprehensive Analysis and Discussion

Based on the above data analysis results, it can be seen that anger reduced the driver’s fixation time on the visual materials in VIT, VWMT and MVIT, and improved the driver’s visual identification accuracy in the VWMT. This result demonstrated that the emotion of anger improves the driver’s visual perception ability, but it does not mean that angry drivers will have better cognitive and behavioral performance. Many previous studies have shown that anger can lead drivers to take risky driving behaviors, which, in turn, adversely affect driving safety [46,47]. However, some scholars have pointed out that anger leads to more frequent aggressive driving behaviors, but does not increase driving errors [48,49]. The findings in this paper may partly explain the phenomenon that anger increases aggressive driving behavior without increasing driving errors. That is, in the state of anger, the body would counteract the driving safety risk due to the expression of anger (risky driving behavior) by improving its own perception ability. At present, there are few research conclusions about the effect of surprise on the driver’s visual perception ability in related fields. The data analysis results in this paper showed that the emotion of surprise increased the driver’s visual material fixation time in the VWMT and MVIT, and reduced the driver’s identification accuracy of visual materials in the MVIT. This indicated that the emotion of surprise reduced the driver’s visual perception ability as a whole. According to the data analysis results, the emotion of fear increased the driver’s fixation time on visual material in the VIT and VWMT, and reduced the identification accuracy of visual materials. The above conclusions support the viewpoint that the emotion of fear increases driving errors proposed by Taylor, J. et al. [50]. The results in this paper that anxiety increases the fixation time on the visual materials in the VIT, VWMT and MVIT, and reduces the identification accuracy of visual materials in the VWMT, are consistent with previous research conclusions that anxiety increases driving errors and augments driving safety risks [51,52]. The effect of helplessness on the visual attention characteristics in the VIT and VWMT in this paper is similar to the emotion of fear, which largely supports the view that helplessness is a negative emotion similar to fear proposed by Fikretoglu, D. et al. [53]. However, it should be pointed out that the influence of helplessness on the visual attention characteristics in the MVIT is different from that of fear. Helplessness increased the driver’s fixation time on relevant visual materials and reduced the visual identification accuracy. According to the data analysis results, the emotion of contempt reduced the driver’s fixation time on visual material in the VIT and MVIT, and improved the cognitive efficiency of the visual materials. However, there are few research conclusions about the influence of contempt on the driver’s visual perception ability in related fields. There was no significant difference in the visual material identification results of the subjects in the emotional states of relief and neuter, indicating that the emotion of relief had no significant effect on the driver’s visual perception. Pleasure is generally considered to be a positive emotion. However, the emotion of pleasure led to the increase in driver’s fixation time on visual materials in the VWMT, and the decrease in driver’s visual identification accuracy in the MVIT. These results are consistent with Dolinski, D. et al.’s view that positive emotions are not necessarily positively related to safe driving [54].

## 5. Conclusions

The visual attention characteristic is the key factor to determine whether the driver can extract important information from the traffic environment and form an effective cognition of the environmental situation. Emotions are a special form of human reflection to objective reality. Drivers with different emotions have significant differences in their feelings, preferences and needs for external stimuli, which, in turn, lead to varying degrees of changes in their perceptual characteristics and cognitive abilities. In this paper, the drivers’ visual attention characteristics for different cognitive tasks and the effects of eight typical driving emotions on drivers’ visual attention characteristics were deeply studied through designing and implementing the Visual Identification Task (VIT), Visual Working Memory Task (VWMT) and Multiple Visual Identification Task (MVIT) in the virtual driving process.

The findings of the present study have several important practical implications for improving the safety and intelligence of the road traffic system. On the one hand, information perception is the basis for the generation of conscious behavior in drivers. Ignoring the influence of the limited visual attention resources on driving behavior would lead to a driving behavior prediction model with the drawback of “taking the driver as absolute rationality”, i.e., holding that drivers have absolutely complete environmental knowledge and powerful computing ability, which is obviously a severe deviation of the reality. The results of this paper reveal the driver’s visual information processing mechanism to a certain extent, which contributes to an accurate understanding of the mechanism of generating driving behavior and can represent an interesting starting point in the improvement of driving behavior prediction. On the other hand, real-time communication of dynamic traffic environment information obtained by in-vehicle information sensing devices (radar, camera, etc.) to the driver is a common mode of assisted driving systems, which can expand the breadth and depth of information perception of the driver during driving. However, it should be noted that the in-vehicle system can seriously endanger driving safety by taking up too many perceptual and cognitive resources from the driver in an inappropriate manner. The results of this paper not only reveal the boundaries of drivers’ perception of different visual cognitive objects, but also examine the stability of drivers’ storage and extraction of visual information. These results can provide a scientific basis for the in-vehicle system to choose the proper time to provide the suitable amount of information to the driver and improve the interaction efficiency and intelligence of the in-vehicle human–computer interaction system. Finally, while many studies on driving emotions have provided evidence that emotions affect driving safety, the mechanisms by which emotions affect driving behavior are not yet clear. Some scholars have concluded that emotions can directly influence driving behavior. Some researchers argued that driving emotions can indirectly affect driving behaviors through driver’s perception, attitude and so on. The results of this study demonstrate the role of eight typical driving emotions on drivers’ visual attentional characteristics. Considering the important role of visual attention in influencing driving behavior, the results of this paper suggest that the influence of emotions on driving behavior is multifaceted and includes at least indirectly influencing driving behaviors through affecting perceptual characteristics. The research results can be applied for the vehicle security warning system and then the accuracy of driving behavior prediction would be improved.

The current study has a few limitations. Firstly, the study is not representative of the entire population, since the participants were recruited using a convenience sample through social media. Nevertheless, recruiting participants in this way is considered a common practice worldwide. Secondly, considering feasibility and safety, data measurements are mostly conducted in specific environments or simulated driving environments, and data test results are susceptible to the influence of the environment. There are some differences between the simulated driving environment and the actual road conditions, and the proficiency of the driver using the driving simulator may also have some influence on the experimental results. Follow-up studies should pay attention to overcome the influence of environmental factors on the study results. Thirdly, static pictures are used to simulate the cognitive objects in driving activities in this paper, which limits the ability to reflect on the complex and changing real-world traffic environment. Designing more relevant experimental methods is the focus of subsequent research.

## Figures and Tables

**Table 1:** Elaboration on the different visual cognitive tasks.

Tasks	Description	Visual Materials	Display Screen
VIT	To identify and report the proportion of vehicle types in visual materials while completing the virtual driving.	Ternary pictures/quintuple pictures/seven-element pictures	Screen 2
VIMT	To identify, report and memorize the proportions of vehicle types in the previous.	Seven-element pictures	Screen 2
VIRT	To identify and report whether the proportion of vehicle types in the subsequent picture is the same as the previous picture.	Seven-element pictures	Screen 2
MVIT	To identify and report the unary pictures on the 3 screens while completing driving in the order from left to right.	Unary pictures	Screen 1/Screen 2/Screen 3

**Table 2:** Explanatory notes of the relevant parameters (symbols) obtained from the experiments.

Collective Name	Symbols	Description
VI	VI3, VI5, VI7	Average fixation time of each participant to each ternary picture, quintuple picture and seven-element picture in the VIT
AVI	AVI3, AVI5, AVI7	Average identification accuracy of each participant for ternary, quintuple and seven-element pictures in the VIT
SVI	SVI3, SVI5, SVI7	Fixation time to identify a basic information unit in a ternary picture, a quintuple picture and a seven-element picture in the VIT
VIM	VIM10, VIM20, VIM30	Average fixation time of each participants to each previous picture in the MTU with the display interval of 10, 20 and 30 s
AVIM	AVIM10, AVIM20, AVIM30	Average identification accuracy of each participant for the previous pictures in the MTU with the interval of 10, 20 and 30 s
SVIM	SVIM10, SVIM20, SVIM30	Fixation time to identify a basic information unit in the previous picture in the MTU with the interval of 10, 20 and 30 s
VIR	VIR10, VIR20, VIR30	Average fixation time of each participant to each subsequent picture in the MTU with the interval of 10, 20 and 30 s
AVIR	AVIR10, AVIR20, AVIR30	Average identification accuracy of each participant for the subsequent pictures in the MTU with the interval of 10, 20 and 30 s
SVIR	SVIR10, SVIR20, SVIR30	Fixation time to identify a basic information unit in the subsequent picture in the MTU with the interval of 10, 20 and 30 s
MulVI	MulVI1, MulVI2, MulVI3	Average fixation time (seconds) of each participant to pictures on screens 1, 2 and 3 in the MVIT
AMulVI	AMulVI1, AMulVI2, AMulVI3	Average identification accuracy of each participant for five unary pictures on screens 1, 2 and 3 in the MVIT

**Table 3:** One-way ANOVA results for SVI.

SVI	SoS ^1^	df ^2^	MS ^3^	F	Sig. ^4^
BG ^5^	0.086	2	0.043	45.172	**0.000**
WG ^6^	0.188	198	0.001		
Tot. ^7^	0.274	200			

^1^ SoS is the sum of squares. ^2^ df is the degree of freedom. ^3^ MS is the mean square. ^4^ Sig. is the significance. ^5^ BG means between groups. ^6^ WG means within groups. ^7^ Tot. means total.

**Table 4:** Multiple comparison results for SVI.

(I)	(J)	MD ^1^ (I–J)	SE ^2^	Sig. ^3^	95% CI ^4^	
					LB ^5^	UB ^6^
SVI7	SVIR10	−0.020 * ^7^	0.004	**0.000**	−0.031	−0.008
	SVIR20	−0.019 *	0.005	**0.000**	−0.031	−0.007
	SVIR30	−0.019 *	0.005	**0.000**	−0.031	−0.007
SVIR10	SVI7	0.020 *	0.004	**0.000**	0.008	0.031
	SVIR20	0.001	0.005	1.000	−0.012	0.014
	SVIR30	0.000	0.005	1.000	−0.012	0.013
SVIR20	SVI7	0.019 *	0.005	**0.000**	0.007	0.031
	SVIR10	−0.001	0.005	1.000	−0.014	0.012
	SVIR30	0.000	0.005	1.000	−0.014	0.013
SVIR30	SVI7	0.019 *	0.005	**0.000**	0.007	0.031
	SVIR10	0.000	0.005	1.000	−0.013	0.012
	SVIR20	0.000	0.005	1.000	−0.013	0.014

^1^ MD is the mean difference. ^2^ SE is the standard error. ^3^ Sig. is the significance. ^4^ CI is the confidence interval. ^5^ LB is lower bound. ^6^ UB is upper bound. ^7,^* means the significance level 0.05.

**Table 5:** One-way ANOVA results for AVI.

AVI	SoS ^1^	df ^2^	MS ^3^	F	Sig. ^4^
BG ^5^	0.067	2	0.034	0.886	0.414
WG ^6^	7.532	198	0.038		
Tot. ^7^	7.600	200			

^1^ SoS is the sum of squares. ^2^ df is the degree of freedom. ^3^ MS is the mean square. ^4^ Sig. is the significance. ^5^ BG means between groups. ^6^ WG means within groups. ^7^ Tot. means total.

**Table 6:** One-way ANOVA results for SVIM.

SVIM	SoS ^1^	df ^2^	MS ^3^	F	Sig. ^4^
BG ^5^	0	2	0	0.166	0.847
WG ^6^	0.152	198	0.001		
Tot. ^7^	0.152	200			

^1^ SoS is the sum of squares. ^2^ df is the degree of freedom. ^3^ MS is the mean square. ^4^ Sig. is the significance. ^5^ BG means between groups. ^6^ WG means within groups. ^7^ Tot. means total.

**Table 7:** One-way ANOVA results for AVIM.

SVIM	SoS ^1^	df ^2^	MS ^3^	F	Sig. ^4^
BG ^5^	0.031	2	0.015	0.288	0.750
WG ^6^	10.630	198	0.054		
Tot. ^7^	10.661	200			

^1^ SoS is the sum of squares. ^2^ df is the degree of freedom. ^3^ MS is the mean square. ^4^ Sig. is the significance. ^5^ BG means between groups. ^6^ WG means within groups. ^7^ Tot. means total.

**Table 8:** One-way ANOVA results for SVI7 and SVIM.

SVIM	SoS ^1^	df ^2^	MS ^3^	F	Sig. ^4^
BG ^5^	0.017	3	0.006	8.159	0.000
WG ^6^	0.186	264	0.001		
Tot. ^7^	0.204	267			

^1^ SoS is the sum of squares. ^2^ df is the degree of freedom. ^3^ MS is the mean square. ^4^ Sig. is the significance. ^5^ BG means between groups. ^6^ WG means within groups. ^7^ Tot. means total.

**Table 9:** Multiple comparison results for SVI7 and SVIM.

(I)	(J)	MD ^1^ (I–J)	SE ^2^	Sig. ^3^	95% CI ^4^	
					LB ^5^	UB ^6^
SVI7	SVIM10	−0.018 *^7^	0.004	**0.001**	−0.030	−0.006
	SVIM20	−0.017 *	0.004	**0.001**	−0.029	−0.006
	SVIM30	−0.020 *	0.004	**0.000**	−0.032	−0.008
SVIM10	SVI7	0.018 *	0.004	**0.001**	0.006	0.030
	SVIM20	0.000	0.005	1.000	−0.012	0.013
	SVIM30	−0.002	0.005	0.998	−0.015	0.011
SVIM20	SVI7	0.017 *	0.004	**0.001**	0.006	0.029
	SVIM10	0.000	0.005	1.000	−0.013	0.012
	SVIM30	−0.003	0.005	0.996	−0.015	0.010
SVIM30	SVI7	0.020 *	0.004	**0.000**	0.008	0.032
	SVIM10	0.002	0.005	0.998	−0.011	0.015
	SVIM20	0.003	0.005	0.996	−0.010	0.015

^1^ MD is the mean difference. ^2^ SE is the standard error. ^3^ Sig. is the significance. ^4^ CI is the confidence interval. ^5^ LB is lower bound. ^6^ UB is upper bound. ^7,^* means the significance level 0.05.

**Table 10:** One-way ANOVA results for AVI7 and AVIM.

SVIM	SoS ^1^	df ^2^	MS ^3^	F	Sig. ^4^
BG ^5^	0.045	3	0.015	0.284	0.837
WG ^6^	13.864	264	0.053		
Tot. ^7^	13.909	267			

^1^ SoS is the sum of squares. ^2^ df is the degree of freedom. ^3^ MS is the mean square. ^4^ Sig. is the significance. ^5^ BG means between groups. ^6^ WG means within groups. ^7^ Tot. means total.

**Table 11:** One-way ANOVA results for SVIR.

SVIM	SoS ^1^	df ^2^	MS ^3^	F	Sig. ^4^
BG ^5^	0.000	2	0.000	0.016	0.984
WG ^6^	0.161	198	0.001		
Tot. ^7^	0.161	200			

^1^ SoS is the sum of squares. ^2^ df is the degree of freedom. ^3^ MS is the mean square. ^4^ Sig. is the significance. ^5^ BG means between groups. ^6^ WG means within groups. ^7^ Tot. means total.

**Table 12:** One-way ANOVA results for AVIR.

SVIM	SoS ^1^	df ^2^	MS ^3^	F	Sig. ^4^
BG ^5^	1.991	2	0.996	14.107	**0.000**
WG ^6^	13.973	198	0.071		
Tot. ^7^	15.965	200			

^1^ SoS is the sum of squares. ^2^ df is the degree of freedom. ^3^ MS is the mean square. ^4^ Sig. is the significance. ^5^ BG means between groups. ^6^ WG means within groups. ^7^ Tot. means total.

**Table 13:** Multiple comparison results for AVIR.

(I)	(J)	MD ^1^ (I−J)	SE ^2^	Sig. ^3^	95% CI ^4^	
					LB ^5^	UB ^6^
AVIR10	AVIR20	0.119 * ^7^	0.043	**0.021**	0.014	0.225
	AVIR30	0.244 *	0.044	**0.000**	0.137	0.351
AVIR20	AVIR10	−0.119 *	0.043	**0.021**	−0.225	−0.014
	AVIR30	0.124 *	0.050	**0.041**	0.004	0.245
AVIR30	AVIR10	−0.244 *	0.044	**0.000**	−0.351	−0.137
	AVIR20	−0.124 *	0.050	**0.041**	−0.245	−0.004

^1^ MD is the mean difference. ^2^ SE is the standard error. ^3^ Sig. is the significance. ^4^ CI is the confidence interval. ^5^ LB is lower bound. ^6^ UB is upper bound. ^7,^* means the significance level 0.05.

**Table 14:** One-way ANOVA results for SVI7 and SVIR.

SVIM	SoS ^1^	df ^2^	MS ^3^	F	Sig. ^4^
BG ^5^	0.019	3	0.006	8.414	**0.000**
WG ^6^	0.196	264	0.001		
Tot. ^7^	0.215	267			

^1^ SoS is the sum of squares. ^2^ df is the degree of freedom. ^3^ MS is the mean square. ^4^ Sig. is the significance. ^5^ BG means between groups. ^6^ WG means within groups. ^7^ Tot. means total.

**Table 15:** Multiple comparison results for SVI7 and SVIR.

(I)	(J)	MD ^1^ (I−J)	SE ^2^	Sig. ^3^	95% CI ^4^	
					LB ^5^	UB ^6^
SVI7	SVIR10	−0.020 * ^7^	0.004	**0.000**	−0.031	−0.008
	SVIR20	−0.019 *	0.005	**0.000**	−0.031	−0.007
	SVIR30	−0.019 *	0.005	**0.000**	−0.031	−0.007
SVIR10	SVI7	0.020 *	0.004	**0.000**	0.008	0.031
	SVIR20	0.001	0.005	1.000	−0.012	0.014
	SVIR30	0.000	0.005	1.000	−0.012	0.013
SVIR20	SVI7	0.019 *	0.005	**0.000**	0.007	0.031
	SVIR10	−0.001	0.005	1.000	−0.014	0.012
	SVIR30	0.000	0.005	1.000	−0.014	0.013
SVIR30	SVI7	0.019 *	0.005	**0.000**	0.007	0.031
	SVIR10	0.000	0.005	1.000	−0.013	0.012
	SVIR20	0.000	0.005	1.000	−0.013	0.014

^1^ MD is the mean difference. ^2^ SE is the standard error. ^3^ Sig. is the significance. ^4^ CI is the confidence interval. ^5^ LB is lower bound. ^6^ UB is upper bound. ^7,^* means the significance level 0.05.

**Table 16:** Multiple comparison results for AVI7 and AVIR.

(I)	(J)	MD ^1^ (I–J)	SE ^2^	Sig. ^3^	95% CI ^4^	
					LB ^5^	UB ^6^
AVI7	AVIR10	0.000	0.038	1.000	−0.100	0.100
	AVIR20	0.119 * ^7^	0.044	0.045	0.002	0.237
	AVIR30	0.254 *	0.045	0.000	0.134	0.373
AVIR10	AVI7	0.000	0.038	1.000	−0.100	0.100
	AVIR20	0.119 *	0.043	0.041	0.003	0.236
	AVIR30	0.254 *	0.044	0.000	0.136	0.372
AVIR20	AVI7	−0.119 *	0.044	0.045	−0.237	−0.002
	AVIR10	−0.119 *	0.043	0.041	−0.236	−0.003
	AVIR30	0.134 *	0.050	0.046	0.002	0.267
AVIR30	AVI7	−0.254 *	0.045	0.000	−0.373	−0.134
	AVIR10	−0.254 *	0.044	0.000	−0.372	−0.136
	AVIR20	−0.134 *	0.050	0.046	−0.267	−0.002

^1^ MD is the mean difference. ^2^ SE is the standard error. ^3^ Sig. is the significance. ^4^ CI is the confidence interval. ^5^ LB is lower bound. ^6^ UB is upper bound. ^7,^* means the significance level 0.05.

**Table 17:** Paired-sample *T*-test results for SVIM and SVIR.

	M ^1^	SD ^2^	SE ^3^	95% CI ^4^		t	df ^7^	Sig. ^8^ (2-Tailed)
				LB ^5^	UB ^6^			
SVIM10-SVIR10	−0.002	0.009	0.001	−0.004	0.000	−1.721	66	0.090
SVIM20-SVIR20	−0.001	0.010	0.001	−0.004	0.001	−1.167	66	0.247
SVIM30-SVIR30	0.001	0.010	0.001	−0.002	0.003	0.590	66	0.557

^1^ M is mean value. ^2^ SD is standard deviation. ^3^ SE is standard error. ^4^ CI is confidence interval. ^5^ LB is lower bound. ^6^ UB is upper bound. ^7^ df is degree of freedom. ^8^ Sig. is significance.

**Table 18:** One-way ANOVA results for MulVI.

SVIM	SoS ^1^	df ^2^	MS ^3^	F	Sig. ^4^
BG ^5^	0.314	2	0.157	26.168	**0.000**
WG ^6^	1.189	198	0.006		
Tot. ^7^	1.503	200			

^1^ SoS is the sum of squares. ^2^ df is the degree of freedom. ^3^ MS is the mean square. ^4^ Sig. is the significance. ^5^ BG means between groups. ^6^ WG means within groups. ^7^ Tot. means total.

**Table 19:** Multiple comparison results for MulVI.

(I)	(J)	MD ^1^ (I–J)	SE ^2^	Sig. ^3^	95% CI ^4^	
					LB ^5^	UB ^6^
MulVI1	MulVI2	0.018	0.013	0.172	−0.008	0.045
	MulVI3	0.092 * ^7^	0.013	**0.000**	0.065	0.118
MulVI2	MulVI1	−0.018	0.013	0.172	−0.045	0.008
	MulVI3	0.073 *	0.013	**0.000**	0.047	0.100
MulVI3	MulVI1	−0.092 *	0.013	**0.000**	−0.118	−0.065
	MulVI2	−0.073 *	0.013	**0.000**	−0.100	−0.047

^1^ MD is the mean difference. ^2^ SE is the standard error. ^3^ Sig. is the significance. ^4^ CI is the confidence interval. ^5^ LB is lower bound. ^6^ UB is upper bound. ^7^* means the significance level 0.05.

**Table 20:** One-way ANOVA results for AMulVI.

SVIM	SoS ^1^	df ^2^	MS ^3^	F	Sig. ^4^
BG ^5^	0.002	2	0.001	0.090	0.914
WG ^6^	1.742	198	0.009		
Tot. ^7^	1.744	200			

^1^ SoS is the sum of squares. ^2^ df is the degree of freedom. ^3^ MS is the mean square. ^4^ Sig. is the significance. ^5^ BG means between groups. ^6^ WG means within groups. ^7^ Tot. means total.

**Table 21:** Paired-samples *T*-test results for VI’ of neuter and VI’ of eight emotions.

VI’	M ^1^	SD ^2^	SE ^3^	95% CI ^4^		t	Sig. ^7^ (2-Tailed)
				LB ^5^	UB ^6^		
neuter-anger	0.029	0.043	0.007	0.016	0.042	4.416	**0.000**
neuter-surprise	0.011	0.068	0.010	−0.010	0.032	1.045	0.302
neuter-fear	−0.173	0.178	0.027	−0.227	−0.118	−6.363	**0.000**
neuter-anxiety	−0.207	0.183	0.028	−0.264	−0.151	−7.449	**0.000**
neuter-helplessness	−0.025	0.051	0.008	−0.041	−0.010	−3.235	**0.002**
neuter-contempt	0.030	0.053	0.008	0.014	0.046	3.695	**0.001**
neuter-relief	−0.015	0.065	0.010	−0.035	0.005	−1.481	0.146
neuter-pleasure	−0.004	0.064	0.010	−0.024	0.016	−0.429	0.670

^1^ M is mean value. ^2^ SD is standard deviation. ^3^ SE is standard error. ^4^ CI is confidence interval. ^5^ LB is lower bound. ^6^ UB is upper bound. ^7^ Sig. is significance.

**Table 22:** Paired-samples *T*-test results for AVI’ of neuter and AVI’ of eight emotions.

AVI’	M ^1^	SD ^2^	SE ^3^	95% CI ^4^		t	Sig. ^7^ (2-Tailed)
				LB ^5^	UB ^6^		
neuter-anger	−0.016	0.071	0.011	−0.037	0.006	−1.431	0.160
neuter-surprise	0.008	0.170	0.026	−0.045	0.060	0.298	0.767
neuter-fear	0.116	0.161	0.025	0.067	0.166	4.743	**0.000**
neuter-anxiety	0.039	0.149	0.023	−0.007	0.085	1.703	0.096
neuter-helplessness	0.047	0.117	0.018	0.011	0.082	2.610	0.013
neuter-contempt	−0.008	0.154	0.024	−0.055	0.040	−0.330	0.743
neuter-relief	0.031	0.142	0.022	−0.013	0.075	1.431	0.160
neuter-pleasure	0.008	0.185	0.028	−0.049	0.065	0.274	0.785

^1^ M is mean value. ^2^ SD is standard deviation. ^3^ SE is standard error. ^4^ CI is confidence interval. ^5^ LB is lower bound. ^6^ UB is upper bound. ^7^ Sig. is significance.

**Table 23:** Paired-samples *T*-test results for VIR’ of neuter and VIR’ of eight emotions.

VIR’	M ^1^	SD ^2^	SE ^3^	95% CI ^4^		t	Sig. ^7^ (2-Tailed)
				LB ^5^	UB ^6^		
neuter-anger	0.035	0.045	0.007	0.021	0.049	5.072	**0.000**
neuter-surprise	−0.019	0.045	0.007	−0.033	−0.006	−2.864	**0.007**
neuter-fear	−0.207	0.185	0.028	−0.264	−0.150	−7.321	**0.000**
neuter-anxiety	−0.191	0.197	0.030	−0.252	−0.131	−6.361	**0.000**
neuter-helplessness	−0.019	0.056	0.009	−0.036	−0.001	−2.185	**0.035**
neuter-contempt	0.010	0.060	0.009	−0.009	0.028	1.070	0.291
neuter-relief	−0.001	0.061	0.009	−0.020	0.017	−0.146	0.885
neuter-pleasure	−0.037	0.047	0.007	−0.051	−0.022	−5.126	**0.000**

^1^ M is mean value. ^2^ SD is standard deviation. ^3^ SE is standard error. ^4^ CI is confidence interval. ^5^ LB is lower bound. ^6^ UB is upper bound. ^7^ Sig. is significance.

**Table 24:** Paired-samples *T*-test results for AVIR’ of neuter and AVIR’ of eight emotions.

AVIR’	M ^1^	SD ^2^	SE ^3^	95% CI ^4^		t	Sig. ^7^ (2-Tailed)
				LB ^5^	UB ^6^		
neuter-anger	−0.070	0.137	0.021	−0.112	−0.028	−3.334	**0.002**
neuter-surprise	0.155	0.168	0.026	0.103	0.207	6.043	**0.000**
neuter-fear	0.155	0.183	0.028	0.099	0.211	5.547	**0.000**
neuter-anxiety	0.078	0.190	0.029	0.019	0.136	2.673	**0.011**
neuter-helplessness	0.070	0.137	0.021	0.028	0.112	3.334	**0.002**
neuter-contempt	0.008	0.212	0.032	−0.057	0.073	0.240	0.812
neuter-relief	−0.031	0.216	0.033	−0.097	0.035	−0.942	0.352
neuter-pleasure	0.016	0.205	0.031	−0.048	0.079	0.496	0.623

^1^ M is mean value. ^2^ SD is standard deviation. ^3^ SE is standard error. ^4^ CI is confidence interval. ^5^ LB is lower bound. ^6^ UB is upper bound. ^7^ Sig. is significance.

**Table 25:** Paired-samples *T*-test results for MulVI’ of neuter and MulVI’ of eight emotions.

MulVI’	M ^1^	SD ^2^	SE ^3^	95% CI ^4^		t	Sig. ^7^ (2-Tailed)
				LB ^5^	UB ^6^		
neuter-anger	0.004	0.008	0.001	0.002	0.007	3.398	**0.001**
neuter-surprise	−0.012	0.016	0.002	−0.017	−0.008	−5.267	**0.000**
neuter-fear	0.001	0.009	0.001	−0.002	0.004	0.916	0.365
neuter-anxiety	−0.038	0.036	0.006	−0.049	−0.027	−6.902	**0.000**
neuter-helplessness	−0.004	0.011	0.002	−0.007	−0.001	−2.327	**0.025**
neuter-contempt	0.004	0.010	0.002	0.000	0.007	2.333	**0.025**
neuter-relief	−0.001	0.013	0.002	−0.005	0.003	−0.722	0.474
neuter-pleasure	0.000	0.013	0.002	−0.004	0.004	0.114	0.910

^1^ M is mean value. ^2^ SD is standard deviation. ^3^ SE is standard error. ^4^ CI is confidence interval. ^5^ LB is lower bound. ^6^ UB is upper bound. ^7^ Sig. is significance.

**Table 26:** Paired-samples *T*-test results for AMulVI’ of neuter and AMulVI’ of eight emotions.

AMulVI’	M ^1^	SD ^2^	SE ^3^	95% CI ^4^		t	Sig. ^7^ (2-Tailed)
				LB ^5^	UB ^6^		
neuter-anger	0.005	0.068	0.010	−0.016	0.026	0.496	0.623
neuter-surprise	0.008	0.089	0.014	−0.020	0.035	0.573	0.570
neuter-fear	−0.008	0.070	0.011	−0.029	0.014	−0.724	0.473
neuter-anxiety	0.021	0.074	0.011	−0.002	0.043	1.838	0.073
neuter-helplessness	0.026	0.053	0.008	0.009	0.042	3.177	**0.003**
neuter-contempt	0.016	0.062	0.009	−0.004	0.035	1.634	0.110
neuter-relief	0.016	0.071	0.011	−0.006	0.037	1.431	0.160
neuter-pleasure	0.028	0.065	0.010	0.009	0.048	2.886	**0.006**

^1^ M is mean value. ^2^ SD is standard deviation. ^3^ SE is standard error. ^4^ CI is confidence interval. ^5^ LB is lower bound. ^6^ UB is upper bound. ^7^ Sig. is significance.

## Data Availability

The data presented in this study are available on request from the corresponding author. The data are not publicly available due to privacy.

## Supplementary Materials

The following supporting information can be downloaded at: https://www.mdpi.com/article/10.3390/ijerph19095059/s1, Figure S1: PAD emotion scale; Table S1: Emotional activation efficacy level corresponding to each point in PAD space.
