# Supplementary material for: Driver’s Visual Attention Characteristics and Their Emotional Influencing Mechanism under Different Cognitive Tasks
Source: Int J Environ Res Public Health. 2022 Apr 21;19(9):5059. doi: 10.3390/ijerph19095059 (PMC9099627; doi:10.3390/ijerph19095059)
Supplement: Supplementary file 1 [file ijerph-19-05059-s001.zip › ijerph-1641959-supplementary.pdf]

Supplementary Material

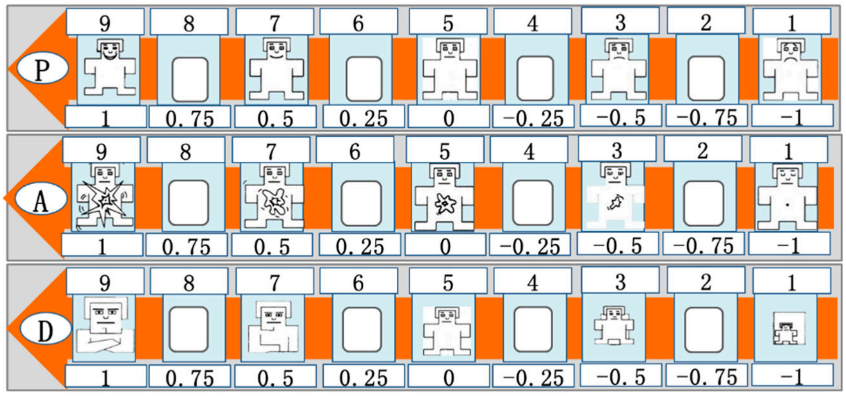

Figure S1. PAD emotion scale

Table S1. Emotional activation efficacy level corresponding to each point in PAD space

Emotion	Distance range	Assigned value	Case distribution[1]	Emotion	Distance range	Assigned value	Case distribution[1]
Anger	0.090~0.496	5	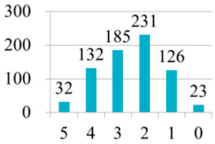	Surprise	0.105~0.421	5	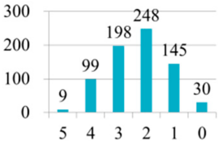
	0.497~0.902	4			0.422~0.736	4	
	0.903~1.307	3			0.737~1.052	3	
	1.308~1.713	2			1.053~1.367	2	
	1.714~2.118	1			1.368~1.682	1	
	2.119~2.524	0			1.681~1.998	0	
Fear	0.164~0.587	5	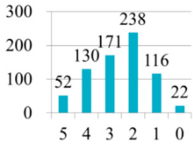	Anxiety	0.121~0.436	5	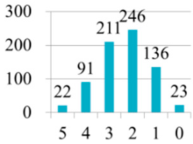
	0.588~1.010	4			0.437~0.751	4	
	1.011~1.433	3			0.752~1.067	3	
	1.434~1.855	2			1.068~1.382	2	
	1.856~2.278	1			1.383~1.697	1	
	2.279~2.701	0			1.698~2.012	0	
Helplessness	0.150~0.492	5	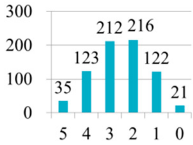	Contempt	0.128~0.469	5	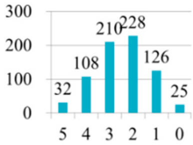
	0.493~0.835	4			0.470~0.809	4	
	0.836~1.177	3			0.810~1.150	3	
	1.178~1.520	2			1.151~1.490	2	
	1.521~1.862	1			1.491~1.831	1	
	1.863~2.205	0			1.832~2.171	0	
Relief	0.122~0.478	5	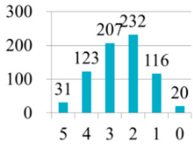	Pleasure	0.150~0.483	5	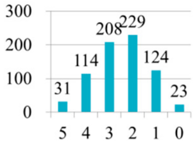
	0.479~0.834	4			0.484~0.816	4	
	0.835~1.189	3			0.817~1.149	3	
	1.190~1.545	2			1.150~1.481	2	
	1.546~1.900	1			1.482~1.814	1	
	1.901~2.256	0			1.815~2.147	0	

[1] The horizontal axis in the case distribution diagram represents the level of emotional activation efficacy, and the vertical axis represents the number of cases.
